# Youth Engaged Participatory Air Monitoring: A ‘Day in the Life’ in Urban Environmental Justice Communities

**DOI:** 10.3390/ijerph17010093

**Published:** 2019-12-21

**Authors:** Jill E. Johnston, Zully Juarez, Sandy Navarro, Ashley Hernandez, Wendy Gutschow

**Affiliations:** 1 Division of Environmental Health, Department of Preventive Medicine, Keck School of Medicine, University of Southern California, Los Angeles, CA 90089, USA; zullyaj@g.ucla.edu (Z.J.); wgutscho@usc.edu (W.G.); 2 LA Grit Media, Los Angeles, CA 90089, USA; sandy@lagritmedia.com; 3 Communities for a Better Environment, Los Angeles, CA 90089, USA; ashley@cbecal.org

**Keywords:** youth, environmental health, citizen science, community science, environmental justice, air pollution, participatory air monitoring, low-cost sensors

## Abstract

Air pollution in Southern California does not impact all communities equally; communities of color are disproportionately burdened by poor air quality and more likely to live near industrial facilities and freeways. Government regulatory monitors do not have the spatial resolution to provide air quality information at the neighborhood or personal scale. We describe the *A Day in the Life* program, an approach to participatory air monitoring that engages youth in collecting data that they can then analyze and use to take action. Academics partnered with Los Angeles-based youth environmental justice organizations to combine personal air monitoring, participatory science, and digital storytelling to build capacity to address local air quality issues. Eighteen youth participants from four different neighborhoods wore portable personal PM_2.5_ (fine particles <2.5 µm in diameter) monitors for a day in each of their respective communities, documenting and mapping their exposure to PM_2.5_ during their daily routine. Air monitoring was coupled with photography and videos to document what they experienced over the course of their day. The PM_2.5_ exposure during the day for participants averaged 10.7 µg/m^3^, although the range stretched from <1 to 180 µg/m^3^. One-third of all measurements were taken <300 m from a freeway. Overall, we demonstrate a method to increase local youth-centered understanding of personal exposures, pollution sources, and vulnerability to air quality.

## 1. Introduction

Ambient air pollution remains an important contributor to global morbidity, especially fine particulate matter (particles <2.5 µm in diameter, PM_2.5_) [1]. Current concentrations of PM_2.5_ continue to adversely affect health, increasing risk of cardiovascular and respiratory disease, as well as neurological disorders [2,3]. Furthermore, living near heavy traffic has substantial adverse effects on lung function development, chronic bronchitis, decreased lung function, heart disease, atherosclerosis, high blood pressure, and birth outcomes [4,5,6,7,8]. Southern California (CA) faces some of the country’s worst air pollution, the nation’s largest port complex, large industrial polluters, and significant traffic pollution. On average, approximately 25–30% of PM_2.5_ in the region is attributed to primary vehicle emissions and ~15% to other mobile sources, as well as a similar contribution from stationary sources (e.g., industrial facilities) [9,10]. Children are particularly vulnerable to the adverse impacts of ambient air pollution, in part due their rapid growth, developmental vulnerability, greater exposure to air pollutants compared with adults, and immature immune systems [11,12,13,14].

Across California and the United States (US), people of color (that is, people who self-identify as Hispanic and/or as a race other than White) in the United States are more likely to live in polluted neighborhoods or near facilities emitting PM_2.5_, a pattern known as environmental injustice [15]. Environmental injustice is highly correlated with other factors that can increase vulnerability to pollution, such as inadequate access to health care, lack of safe spaces to play, lack of access to healthful foods, low-wage jobs, poor-quality housing, and high prevalence of violence [16]. Vulnerable and marginalized communities often carry a legacy of mistrust of government agencies to identify and address neighborhood-scale exposures and often have less access to resources to conduct environmental monitoring [17,18,19]. Identification of environmental hazards and human exposure can be highly valuable information for self-protection, for pollution prevention, and for remediation—issues that are all of concern in communities facing environmental injustice [20]. Increasingly, community organizations and residents are calling for a greater role in scientific research and decision-making that impacts their lives, as well as tools to document such exposures. Environmental justice organizations, that is, groups organizing to address the disparate burden of environmental hazards in working poor communities of color, are using research tools to collect and interpret their own data, offering opportunities to democratize the research paradigm [21,22,23,24,25].

The paradigm of air pollution monitoring is shifting to lower-cost, easier-to-use, portable sensors that can provide high-resolution data in real time [26]. Regulatory air monitoring systems generally do not assess air quality at a spatially refined or neighborhood level [27]. The increase in the availability of low-cost air pollution sensors has increased the number of community residents collecting and using air quality data to better characterize and understand their local environment [28]. Portable, low-cost sensors offer the potential to expand the temporal and spatial coverage of air quality information [29,30,31] and increasingly are being used in exposure and health studies [32,33,34]. These sensor technologies offer new opportunities in urban environmental justice communities to gather neighborhood-level data that illustrate the impact of emissions sources and/or the magnitude of air quality issues affecting their communities To date, there is limited research on how or why sensors and sensor data are used by communities [35].

The Southern California (CA) region is marked by poor air quality; and communities of color are disproportionately burdened by poor air quality and more likely to live near freeways or other industrial facilities [36,37]. Air pollution from industrial facilities and roadways, in particular, plays a significant role in causing a variety of health problems among children [38,39,40,41,42]. Studies in the US have demonstrated that black and Latinx children live and/or go to school near more sources of air pollution [43], closer to freeways [44,45], or near industrial agriculture operations [46]. In Los Angeles, it was found that Latinx students are significantly more likely to attend schools near environmental hazards [47]. However, these studies rely on distance metrics or exposure modeling rather than actual measurements to assess exposures.

**Figure 1:**
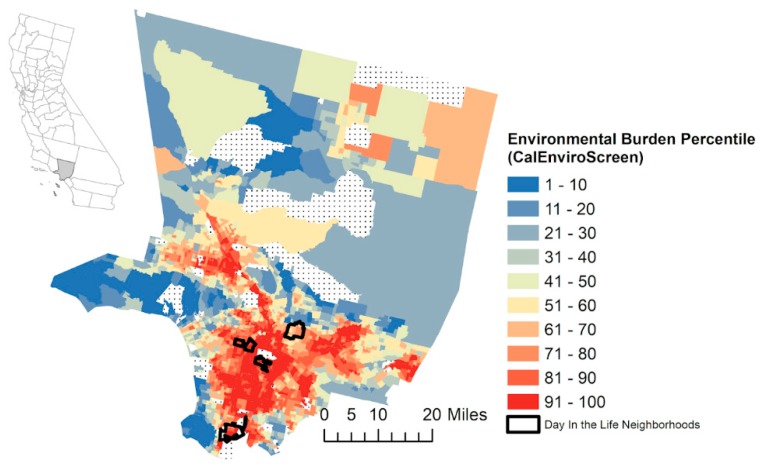
Neighborhoods of youth participants in the *A Day in the Life* program in Los Angeles County. Shown with the percentiles for environmental pollution and community vulnerabilities by census tract as measured by the CalEnviroScreen 3.0 tool from the state of California [48]. Percentiles are calculated based on exposure indicators (air quality, diesel particulate matter, drinking water contaminants, pesticide use, and toxic releases from facilities and traffic density), environmental effect indicators (cleanup sites, groundwater threats, hazardous waste generators and facilities, impaired water bodies, and solid waste sites and facilities), sensitive population indicators (asthma, cardiovascular disease, and low birth weight infants), and socioeconomic factor indicators (educational attainment, housing burden, linguistic isolation, poverty, and unemployment).

We describe a collaborative participatory personal air monitoring and storytelling program between the University of Southern California Community Engagement Program on Health and the Environment (USC CEPHE) and three environmental justice organizations grounded in different neighborhoods in Los Angeles County: Communities for a Better Environment (CBE), South Central Youth Leadership Coalition (SCYLC), and Promoting Youth Advocacy/Asian and Pacific Island Forward Movement (PYA). This is a community-driven pilot project to leverage portable, low-cost sensor technology to assess exposure to PM_2.5_ by youth living among the communities most disproportionately burdened by environmental hazards in the state, based on CalEnviroScreen 3.0 (Sacramento, CA: California Environmental Protection Agency, 2018) [38] (Figure 1). CalEnviroScreen is a screening tool at the census tract level for the state of CA that evaluates the burden of pollution from multiple sources in communities while accounting for potential vulnerability to the adverse effects of pollution. All four neighborhoods are among the top 15% of communities most burdened by environmental hazards in the state (that is a CalEnviroScreen score >85th percentile). Communities for a Better Environment (CBE, http://www.cbecal.org/) builds people power in low-income communities of color to achieve environmental health and justice by preventing and reducing pollution and building green, healthy, and sustainable communities. CBE organizes residents in Huntington Park, a predominantly low-income Latinx city along a goods movement truck route and situated adjacent to industrial operations. Similarly, Wilmington, another community where CBE organizes, is home to a high percentage of Latinx and foreign-born residents and incudes a heavy concentration of industry, including the Port of Los Angeles. SCYLC is a grassroots coalition of youth whose mission is to work collaboratively with allies to advocate for the health, safety, and human rights of communities living near freeways and urban oil drilling sites. SCYLC works with youth of color in the North University Park neighborhood in the City of Los Angeles, which is intersected by two freeways and located near multiple oil extraction wells [37]. Promoting Youth Advocacy (PYA) is a student club that works collaboratively with Asian and Pacific Island Forward Movement (APIFM, https://www.apifm.org/) to address a range of community health and environmental justice issues. The community is approximately 53% Asian/Asian-American and 35% Latinx. The PYA students attend a high school in Alhambra, CA, adjacent to the I-10 freeway, which carries thousands of cars and trucks daily.

*A Day in the Life* is a community-driven participatory air monitoring program with a focus on personal exposure, air pollution measurements, and youth engagement. This program combines low-cost air monitors with multimedia tools to increase the capacity of local youth and environmental justice organizations to use and leverage air quality data and low-cost sensors to communicate about public health impacts through storytelling. The program was co-created among academic, organizational, and youth partners to develop the questions, project design, data collection, data analysis, and production of knowledge. The aim was to increase environmental health literacy, collect community-owned data, and combine these resources with telling stories through digital media to promote awareness about personal exposures to PM_2.5_ among high school youth of color living in environmental justice communities urban Los Angeles County, CA.

## 2. Materials and Methods 

The *A Day in the Life* program was implemented using a four-phase strategy to build the capacity of youth leaders to understand the sources of air pollution and its relationships to health, develop technical skills with respect to air monitoring and storytelling, facilitate critical conversations to assess air monitoring results, and communicate the results back to their communities through personal narratives. The program integrated popular education-based techniques that built upon local concerns and leveraged knowledge based on personal experience to provide a foundation to understand air pollution and links to health effects. The design of the program is described below. 

*Phase I: Popular Education Workshops.* The program, launched in each partner organization with an interactive workshop, covered a broad overview of air pollution with a focus on fine particulate matter, PM_2.5_. The workshop equipped youth to understand PM_2.5_: its sources, regulatory framework, how it is measured, units of measurement, health effects, and how PM_2.5_ differs from other air pollutants (e.g., diesel particulate matter). The second part of this workshop was a skill building training on digital storytelling by LA Grit Media, a local organization working at the intersection of media and social justice by amplifying and archiving community stories to advance local social movements. The participants learned skills with respect to capturing camera shots and using a storyboard format. The concept of storytelling was then integrated into individualized planning of their personal narrative with respect to their daily exposures to PM_2.5_. 

*Phase II: Measuring Air Quality.* The AirBeam (HabitatMap, Brooklyn, NY, USA) [49] is a low-cost, portable instrument that uses a light scattering method to determine PM_2.5_ concentrations (μg/m^3^) in ambient air [50,51]. A light-emitting diode is used to detect particles; the raw measurement provides particle counts, which is converted to mass concentration based on assumptions about the particle mass density, the refractive index, and the size distribution [52]. Air quality measurements are recorded every second via Bluetooth to the AirCasting Android application (AirCasting v1.5.10, HabitatMap, Brooklyn, NY, USA, http://aircasting.org), an open source firmware developed specifically to be used with the AirBeam instrument. AirBeam sensors have demonstrated high precision in the field and are reasonably correlated with reference instruments [53]. However, the AirBeam can exhibit a negative bias when compared with regulatory instruments, especially at elevated PM_2.5_ concentrations [54]. Organizational staff and youth participants were trained on how to use the monitor, including a participatory activity that gave them hands-on experience and prepared them to use the monitor on their own. The application is capable of recording, displaying, and sharing time series of personal PM_2.5_ concentrations, temperature, and relative humidity. Each AirBeam was paired with a Samsung Galaxy J3 Emerge phone (Android 6.0.1, Samsung Electronics America, Ridgefield Park, NJ, USA), which captured the geolocations every second. Each pair was provided to the participants for 4–5 days to record their *Day in the Life* personal exposure measurements. The real-time instantaneous exposure data can be viewed on the smartphone. As part of this process, a technical manual on using the AirBeam and AirCasting app was developed to support youth and staff using the sensor in the field [55]. Data were uploaded to the web-based AirCasting crowdsourced air quality map [56].

In the first cohort, high school youth members of the three partner organizations, CBE, SCYLC, and PYA, were recruited by the respective organizations to participate in the fall of 2017. The youth provided written permission from an adult guardian. During air monitoring sessions, the youth documented what they saw and observed through photographs, videos, and a written journal. The goal was to capture at least 8–10 h of data on a single day to provide a snapshot of air quality during their daily routines, such as at school, at work, and while commuting. The youth wore the device using a lanyard or by attaching the device to their backpack or purse. 

*Phase III: Reflection, Analysis, and Interpretation.* The youth each received processed air quality data and maps prepared by USC CEPHE. During the reflection workshop, the youth met with USC CEPHE and organizational staff to share their experiences using the sensors and to discuss new questions or ideas that arose from the project. Each youth shared their map, reflected on the data and the difference in exposures during the day, and offered critical insights about air quality. From this process emerged new questions and reflections upon what the sensor captured, what types of air pollution sources were measured, and how this related to the hypothesis each youth generated. The youth provided oral and written reflections about their experiences in groups or individually.

*Phase IV: Youth Community Forum and Action.* As a culminating component of the program, youth from all the organizations participated in a forum with their peers and community members. The Los Angeles Youth for Environmental Justice, or #LAYouth4EJ, forum featured *A Day in the Life* projects and highlighted youth voices in the context of addressing air pollution.

*Data Analysis.* After an AirBeam session was recorded on the associated phone and the session was synced, USC CEPHE reviewed all the recorded air quality monitoring sessions and compiled recorded sessions of each youth participant. Data from all sessions were also downloaded, cleaned, and mapped using SAS 9.4 (SAS Institute, Cary, NC, USA) and JMP 13 (SAS Institute, Cary, NC, USA). A map of each program participant’s route showing their PM_2.5_ exposures on a single day was then printed on poster-sized paper so that the youth could look at the PM_2.5_ concentrations across space and time and tell their narrative story about their mapped day. The youth participant testimonies were recorded and transcribed to identify patterns and themes around knowledge, risk, values, and beliefs, using a content analysis approach. Descriptive statistics of PM_2.5_ concentrations and spatial patterns of concentrations were analyzed with ArcGIS (ESRI, Redlands, CA, USA) and STATA IC 14 (College Station, TX, USA).

## 3. Results

“I decided to join this project because I was able to describe my story and show myself how affected I am by pollution. It is important to me because I want to share my story to open the eyes of others. Where I live, not a lot of people know there is an oil well. People don’t question their surroundings, they should know.”—SCYLC youth, South Central Los Angeles

“I wanted to talk about the different types of transportation in my community. I live in Huntington Park [HP]. Living in HP we are surrounded by five freeways, those five freeways bring in diesel through trucks that transport things that we use on a daily basis. We live really, really, really close to the City of Vernon where all the factories are.”—CBE youth, Huntington Park

“I value my community. It’s my home. It’s where I am growing up. Although I believe there isn’t a pretty view of it and it’s not a very safe or healthy environment, I will always remember Wilmington. I began getting really involved in environmental justice work when my younger sister was born with a heart condition. My sister is really the one that motivates me to continue to be involved in the community, and the desire to change Wilmington for the better.”—CBE youth, Wilmington

### 3.1. Participants 

Eighteen youth participants from three community-based organizations completed the initial *A Day in the Life* program. Of these, 13 were female. Fifteen identified at Latinx and three as Asian-American. The youth all attended high school and ranged in age from 15 to 17 years old. They attended nine different high schools. In total, the youth recorded 340 h of data in over 120 sessions, with an average of 18 h per participant. Unfortunately, only one of five youths from SCYLC completed the monitoring, because noise, rather than PM_2.5_, was recorded by the other four youth participants. In the end, 10 out of the 18 youths were able to capture consecutive data to represent a complete day (that is waking hours) with at least one and often multiple recorded sessions within a single day.

*Youth-Driven Questions and Motivations*. The youth participants brought up several potential sources of PM_2.5_ that they felt could affect them at home or at schools, including trucks and heavy freeway traffic, the activities at the Port of Los Angeles and Long Beach, oil extraction sites, and petroleum refineries. The youth all lived in neighborhoods, that, according to a state tool to assess cumulative burden to environmental exposures, ranked among the top 15% of most environmentally burdened neighborhoods in the state of California [48].

Prior to conducting monitoring, each youth designed a question that could be addressed using personal PM_2.5_ sensors. Several youths shared that they spend the majority of their day at school and were interested in characterizing PM_2.5_ both inside and outside of the buildings. One focused on exposure at nearby parks. Others were interested in their neighborhood and home exposures, and one compared PM between her neighborhood and one with fewer industrial activities. Students brought up questions about exposure during bus rides or walks to and from school. 

### 3.2. Air Monitoring Results 

“[I noticed] school has cleaner air, what I noticed from the AirBeam monitor is that it doesn’t fluctuate a lot, it mostly stays in the green but obviously for PM there is no safe level to breathe in. In the regular classroom it was no more than 10 µg/m^3^ …. Outside of my school for swim class, the PM will begin to change from 15 to 20 µg/m^3^. Once the monitor shot up to 70 to 80 µg/m^3^ because our janitor has a small car and it releases so much gas.”—CBE youth, Huntington Park

“At my school you can see spots above 12 µg/m^3^, many [air pollution spikes] happening around the gym and there is a freeway over there. At school the PM peaked at 95 µg/m^3^ by the pool…”—PYA youth, Alhambra

“What I found interesting about my Day in the Life is at home my mom cooks a lot with the windows closed, the PM went up to 28 µg/m^3^. At school I think they are doing something to not have PM because it was 0 or 1 µg/m^3^, then I walked to McDonald’s and it was 67 to 70 µg/m^3^… As I wait outside all the pollutants are coming out of the trucks, factories and the Farmer John slaughterhouse and we all smell it here at school and at Huntington Park.”—CBE youth, Huntington Park

**Figure 2:**
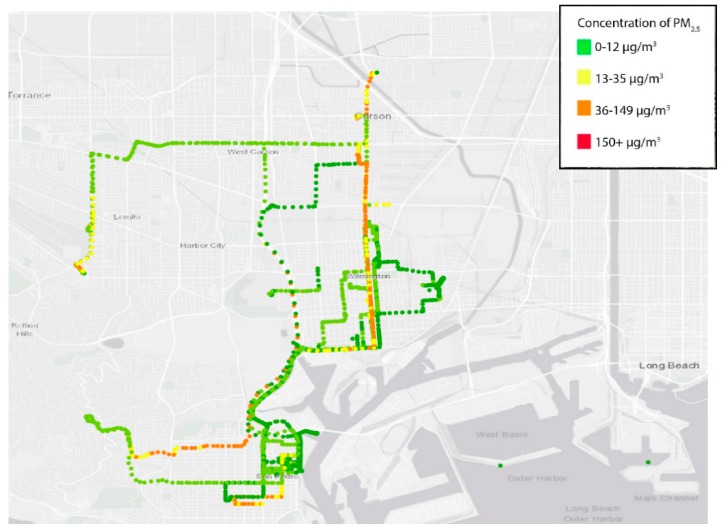
Map of PM_2.5_ air monitoring exposure measurements from all CBE youth participants.

The results were averaged into 10 s observations for subsequent reporting and analyses (Table 1) and shown across space (Figure 2). Typically, the PM_2.5_ exposure during the day for the participants was 10.7 µg/m^3^, although the range stretches from <1 to over 180 µg/m^3^. A total of 3 out of the 10 participants had average exposures above 12 µg/m^3^, the California Environmental Protection Agency health protective standard for PM_2.5_. Five youths recorded measurements above 35 µg/m^3^ for at least 5% of the time (up to 14%). Approximately one-third of all measurements were taken less than 300 m from a busy freeway. Three students spent the majority of their day near a freeway (69–93% of the time). 

**Figure 3:**
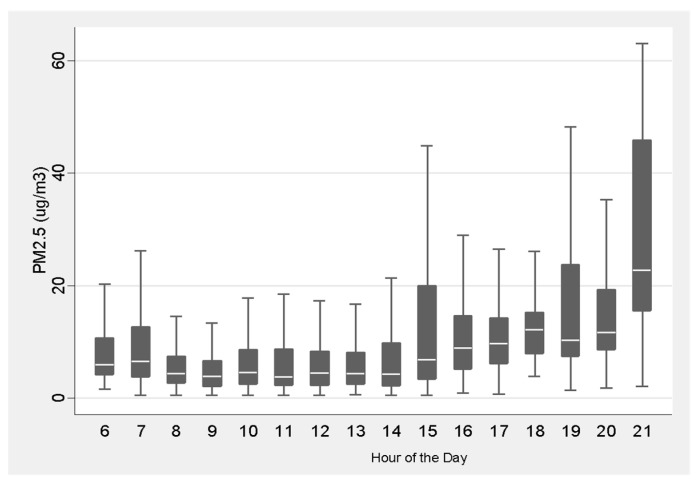
Summary box plots of PM_2.5_ concentrations by hour (ug/m^3^) of the day for participants. Typically, the participants are in school from about 08:00–15:00. More variability and higher exposures are typically observed in the late afternoon and evening hours.

Overall, exposures were highest after school hours (after 15:00) for the participants (Figure 3), which may be due to after-school outdoor sports activities, as well as proximity to indoor cooking. 

**Figure 4:**
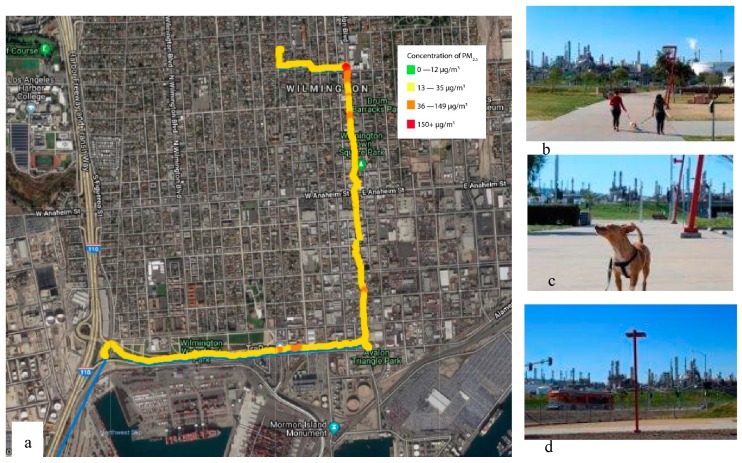
An example of the combination of images (all taken by the participant) with sensor data. (**a**) Map of youth air monitoring route recorded with AirBeam on AirCasting app. (**b**) A park adjacent to an oil refinery complex in Wilmington, CA. (**c**) The participants dog walking at the park. (**d**) A view of the industrial complex from the park. The participant describes the following: “I took part in a program with University of Southern California to help track the air quality in my community. After walking around Wilmington and San Pedro with an air monitor, I was surprised with the results. On the map shown, you are able to see one of my routes to school. The yellow and orange indicate the higher levels of fine particulate matter in the air. This was shocking to see. I decided to focus my images on the pollution happening around our parks. These are only two parks that are near my home, where the refineries are visible, and you can see the particulate matter in the air. Many babies, kids, adults, and even animals breathe in this pollution that causes serious damage to one’s health. That is why I am in CBE [Communities for a Better Environment], to make a difference in my community and to stop this from getting any worse.”

For each youth, a StoryMap (an interactive web-based geospatial, ESRI, Redlands, CA, USA) of their route, along which they measured their air pollution exposure, was created and made available publicly [57]. An example of a participant’s map and photographs is illustrated in Figure 4.

## 4. Discussion

“With this project I imagine what will happen if I will be exposed to this [PM_2.5_] day by day, I imagine how PM may have an effect [on] our health, research shows it has a relation, and how a little as one air monitor test can make a difference, everyone here can make a difference as well.”—PYA youth, Alhambra

“I was able to tell my family about this project, I also presented at my environmental engineering class where I talked about my experience with the AirBeam and how along the refineries the particulate matter was really high 40, 50 [micrograms per cubic meter] and how bad it is for us in our community to breath all this air because it can cause potential damages such as headaches, nosebleeds, asthma etc. I want to take action and hopefully share this with a lot more people to show how it is affecting our community.”—CBE Youth, Wilmington

“I enjoyed doing this project because it was a lot of new information for me that directly impacts me as a community member, as well as learning about the way particulate matter affects our daily lives. With all of this new information, I want to educate my community on how harmful these particulates are, and how change should begin with personal choices people make throughout their day.”—CBE Youth, Huntington Park

“This project made me feel that I want to spread the word, I want people to know what is going on in our community, they deserve to know and take part in what we are doing to stop oil drilling.”—SCYLC youth, South Central LA

The youth participants live in communities characterized by environmental injustice, that is, facing an inequitable and disproportionate burden of environmental health threats, coupled with fewer resources and limited polity [36]. Participatory air monitoring with low-cost sensors can build upon principles of community-driven participatory research, which seeks to deconstruct traditional power dynamics, provide information about environmental hazards important to residents, and democratize knowledge [58]. This is an example of an approach to bridge the gap between increasingly technical air monitoring data and community expertise and knowledge. Further, the process of engaging in participatory air monitoring can increase capacity for youth to engage in environmental health and encourage participation in the political and regulatory systems [59].

Assessing personal exposure to air pollution gives a more realistic assessment of the individuals’ spatiotemporal exposure in comparison to government monitors, especially with respect to indoor and commuting exposures. Personal monitoring offers an opportunity to characterize human exposure at a granular scale relevant to residents and understand localized spatiotemporal factors influencing PM_2.5_ exposures. The project leveraged local youth knowledge to design a project consistent with their daily life priorities and experiences. The coupling of hands-on activities with storytelling aimed to develop a sense of ownership over the data and the project, which is key to the sustainability of not only the project but also, more broadly, skills in leadership, critical thinking, and environmental health literacy [60,61]. 

Youth engagement in air monitoring at the local level, particularly youth of color from working class communities, can build a foundation for action designed for distinct neighborhoods and foster efforts to create a healthier environment [62,63]. 

*Sensor and Environmental Health Literacy*. Environmental health literacy describes the learning and agency involved in growing an understanding of the role of environmental hazards on health, which can translate into improvement of health outcomes for an individual or community [61]. The process of engaging with air quality monitoring can build capacity to partner with academics, as well as developing actions to reduce exposures [35]. During the course of this project, youth participants engaged in activities relevant to the Environmental Health Literacy Triangle set forth by Bloom and built upon by others [61,64]. 

**Figure 5:**
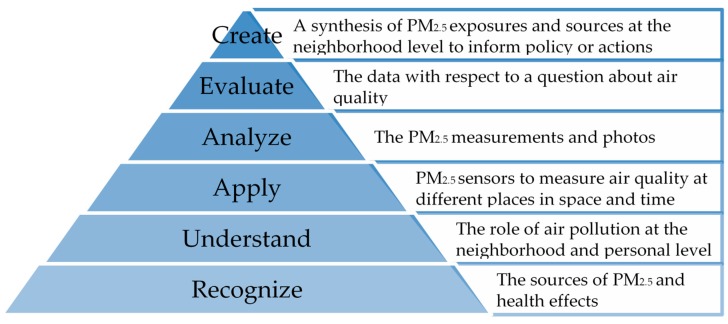
Application of the Environmental Health Literacy Triangle [61] to the *A Day in the Life* program.

During the first phase of the program, during which the youth learned about air pollution and were trained to use the air quality sensor, they developed a recognition and understanding of air pollution and how it relates to their communities—the two foundational levels of environmental health literacy (recognize and understand, Figure 5). The youth then demonstrated their understanding of the content by identifying air pollution hazards in their communities with each other, with workshop leaders, and with their community organizers or teachers. Next, the youth then applied their knowledge about air pollution and monitoring through their organization and analysis of their mapped data and communicated about their community-based air monitoring experiences. The youth were able to share experiences and lessons learned with their families and communities in public meetings and a national conference. The youth analyzed the data that they had collected that documented how PM_2.5_ is characterized throughout their communities.

Furthermore, through the experience of collecting data and documenting their day in photos and videos, the youth analyzed air pollution data together with their daily realities. Through this process, the youth were able to evaluate their experiences and form new questions that they could seek to answer with respect to their exposures to other air pollutants and air toxins in their communities. Based on their air pollution knowledge and monitoring experience, the youth demonstrated their ability to engage at multilevel stages of the Environmental Health Literacy Triangle through the development of new community-based air monitoring questions and activities. Such activities may further serve as a tool to document previously identified environmental problems, conduct ground-truthing of neighborhood air quality, or validate external sources of air quality data [65].

*PM Sensors in Cumulatively Burdened Urban Communities: Limitations.* Measuring only PM_2.5_, a primarily regional pollutant, fails to capture the complexity of air quality in urban Los Angeles and the range of exposure experienced by the participants. The AirBeam has been shown to be sensitive to particle types [54] and relative humidity levels [66]. This demonstrates the need to consider other environmental factors that may influence exposure measurements when doing the interpretation. The youth had a strong interest in assessing the impact of truck-related traffic; however, the proximity to traffic was not always related to elevated levels of PM_2.5_. This presented an opportunity, however, to capture the limitation of sensor technology and introduce ultrafine particles and diesel pollution. It is difficult to discern indoor versus outdoor time; however, this tool offers the potential to examine commuting patterns and exposures during the school day. Personal air monitors have the potential to transfer responsibility from emissions reductions to vulnerable populations themselves who feel a responsibility to reduce exposure [35]. However, the workshops contextualized both personal choices and policies that could reduce exposures. 

When collaborating in vulnerable communities, it is important to note that the mapping relied on GPS tracking that was available on a public-facing website [56]. It is critical for the youth and their parents/guardians to understand this and to safeguard the personal identify and the individual air monitoring routes recorded by each youth. In addition, as seen by the maps and noted by the participants, the geolocating was not always accurate indoors. However, with the participants’ documenting notes, observations, and photographs on location, time, traffic, and weather, we were able to understand the data in more detail. Our experience demonstrates the importance of integrating monitoring devices with the capacity building of youth in order to increase their environmental health literacy and experience meaningful engagement in their environment as it relates to their exposures to air pollution and how they relate those exposures to their lived experiences through storytelling.

## 5. Conclusions

“I am surrounded by factories, refineries, and ports. I breathe the toxins that go in and out of my body every day. Low-income communities not only face discrimination because of their skin but they also face environmental racism.”—CBE youth (Wilmington)

Participatory air pollution monitoring activities can help increase environmental health literacy, raise awareness, inform policymakers, and contribute to decisions to improve public health. Prior work suggests that the sensor itself is only the beginning of a process to understand and respond to personal air pollution exposure and collect supplementary information that is key to interpreting the data [64]. The *A Day in the Life* project demonstrates a replicable participatory monitoring of personal exposure to air pollution that combines the science of data collection, the skills of leveraging the art of storytelling, and interpretation with actionable information about the realities of air pollution for youth living in cumulatively burdened communities. Our project developed tools to engage youth living in environmental justice communities in air pollution assessment using participatory air monitoring through primary data collection and intensive trainings. This structured program integrated personal air monitoring with building capacity, environmental health literacy, technical skills, storytelling skills, and collaboration between scientists, youth, and environmental justice organizations.

## Figures and Tables

**Table 1:** Summary data from *A Day in the Life* participants.

Youth *	Organization	Minutes	Mean PM_2.5_ (μg/m^3^)	Standard Deviation (μg/m^3^)	% of Time PM_2.5_ Exposure >12 μg/m^3^ **	Minimum (μg/m^3^)	Maximum (μg/m^3^)
A	CBE	7547	8.55	13.52	11.1	0.59	96.62
B	SCYLC	7677	8.25	7.64	17.1	0.77	92.36
C	CBE	8111	6.02	10.21	12.8	0.54	62.05
D	CBE	5686	8.00	9.08	19.3	0.61	110.09
E	CBE	15,002	17.97	33.43	33.2	0.55	186.85
F	CBE	7853	15.89	17.58	45.2	0.54	120.96
G	PYA	12,382	8.99	11.10	13.6	0.77	89.14
H	CBE	8779	7.23	9.75	10.2	0.74	114.99
I	PYA	14,748	13.10	21.83	22.9	0.98	128.82
J	PYA	6932	4.59	2.54	2.1	1.24	50.43
All		94,717	10.73	18.82	20.0	0.54	186.85

* PM_2.5_: fine particulate matter <2.5 µm in diameter); μg/m^3^: Micrograms per cubic meter CBE: Communities for a Better Environment; SCYLC: South Central Youth Leadership Coalition; PYA: Promoting Youth Advocacy. ** The percent of time the youth’s exposure exceeded the California Environmental Protection Agency health protective standard for PM_2.5_ of 12 micrograms per cubic meter (μg/m^3^).
